# Image-guided laser ablation in the treatment of recurrence of renal tumours: technique and preliminary results

**DOI:** 10.1186/s41747-019-0127-0

**Published:** 2020-01-03

**Authors:** Federica Ferrari, Giovanni Mauri, Luca Nicosia, Gianluca Maria Varano, Guido Bonomo, Franco Orsi

**Affiliations:** 1 Postgraduate School in Radiodiagnostics, Università degli Studi di Milano, Milan, Italy; 2 Division of Interventional Radiology, European Institute of Oncology IRCCS, via Giuseppe Ripamonti 435, Milan, Italy; 3 Department of Oncology and Hematoncology, University of Milan, Milan, Italy; 4 Division of Breast Radiology, European Institute of Oncology IRCCS, Via Giuseppe Ripamonti 435, Milan, Italy

**Keywords:** Abdomen, Carcinoma (renal cell), Kidney neoplasms, Laser therapy, Neoplasm recurrence (local)

## Abstract

Abdominal recurrences of renal cell carcinoma (RCC) after surgery might represent a challenge for treatment, often requiring difficult surgeries or anticipated systemic therapy. Our aim is to illustrate a novel application of laser ablation for the treatment of abdominal recurrences of RCC. Patients with abdominal recurrences of renal cancer were treated under ultrasound/computed tomography guidance with a diode laser inserted into the lesion through a thin 21-G needle. A fixed 3-W power protocol was used, changing the illumination time according to lesion dimension and shape. Also, technical success, technical efficacy, local tumour progression, and major and minor complications were retrospectively analysed. Three patients were treated with image-guided laser ablation for abdominal recurrences of RCC. In all cases, it was possible to perform ablation as preoperatively planned and all three nodules (size of 6, 8, and 12 mm) were completely ablated with no evidence of residual enhancement after 6 weeks at contrast-enhanced CT. No minor or major complications were observed. No local tumour progression was reported up to 12 months from ablation. Image-guided laser ablation holds the potential to offer a minimally invasive treatment to patients with abdominal recurrence of RCC. Further studies are needed to evaluate the clinical role of this technique.

## Key points

- Three patients with abdominal recurrence of renal tumours underwent image-guided laser ablation.
- All three treatments were successful, without any complications.
- No local tumour progression was reported up to 12 months from ablation.
- Difficult reoperation could be avoided and systemic therapy could be postponed by successful laser ablation of abdominal recurrences of renal tumours.

## Background

Renal cell carcinoma (RCC) is a common urologic malignant lesion in adults and accounts for 2.6% of all cancers [1]. It may occur at any age, although most patients are older than 40 years, and people in the seventh and eighth decades of life are most commonly affected. Other risk factors are smoking, obesity, and hypertension [2].

Surgery, in the form of radical or partial nephrectomy, is still considered the first choice of treatment for RCC [3], while image-guided thermal ablation is rapidly emerging as an effective alternative, with similar results, lower complications and lower costs [4–7]. However, while most of the patients benefit from these treatments and remain disease-free for years, approximately 2–4% of cases can relapse [8]. Treatment options are limited for locally recurrent RCC, and there are currently no consensus treatment protocols.

Image-guided thermal ablations have been proposed also for the treatment of recurring lesions with the aim of achieving an accurate and precise eradication of the target tumour with a reduced invasiveness. Some studies in limited numbers of patients have reported promising results with radiofrequency ablation (RFA) of local relapses of RCC [9–11].

Laser ablation is the ablative method with the smallest applicator among various ablative techniques and, for this reason, has been proposed as a theoretically ideal ablative modality for the treatment of small lesions in difficult locations [12–15]. Thus, we developed a novel treatment strategy, implying ultrasound and CT guidance for the application of laser ablation to abdominal recurrences of RCC, in order to minimise the invasiveness of the treatment.

The aim of the present paper is to illustrate our treatment technique of laser ablation for the treatment of recurrences after renal surgery in patients treated for RCC.

### Patients and technique

All cases were discussed in our multi-disciplinary team, composed of one urologist, one oncologist, and one interventional radiologist. Image-guided thermal ablation was proposed as the best treatment option by consensus. All patients were visited by one interventional radiologist in the outpatient consultation room, and a full explanation of the procedure was given with particular attention to the pros and cons. Thus, a formal written informed consent was obtained. Patients also provided written informed consent for the use of clinical data for research purposes. The internal institutional review board approved the retrospective study. A week before the treatment, all patients were referred to our preliminary assessment service for a complete check of their general clinical condition by an anaesthesiologist.

Patients’ characteristics, staging for the primary lesion, and type of primary treatment are reported in Table 1.

**Table 1:** Patients’ characteristics

	Patients		
	1	2	3
Age	42	69	73
Sex	M	M	M
Tumour histopatology	RCC	RCC	RCC
Staging	pT1b pNx	pT3a pN0	pT1 pNx
Surgical treatment	Radical nephrectomy	Radical nephrectomy	Enucleoresection

*RCC* Renal cell carcinoma

Image-guided laser ablations were performed in a dedicated operatory room equipped with CT and ultrasound machines, under general anaesthesia. Patients were positioned choosing the most favourable path for a direct needle approach to the lesions [4]. Also, CT and ultrasound real-time fusion imaging was available and applied during the procedure [4]. A preliminary CT was performed with the patient in the chosen position, and the CT dataset was used for fusion with real-time ultrasound. Then, a 21-G needle was inserted under ultrasound monitoring at the level of the lesion to be treated, and its correct position confirmed by an unenhanced CT acquisition. If needed, hydrodissection through the injection of sterile water was also performed before ablation in order to protect adjacent structures from heat damage [4]. Then, a 0.3-mm laser fibre was inserted into the 21-G needle and thermal ablation performed with a commercially available semi-conductor diode laser system with a wavelength of 1064 nm (Echolaser, Elesta Srl, Florence, Italy). A fixed 3-W power protocol was used with a total energy delivered of 1,200–1,800 J for a single illumination. At the end of the procedure, a triple-phase contrast-enhanced CT scan (acquisition of arterial, venous and late phase, with an injection rate of 3.5 mL/s) was performed to evaluate the immediate result of ablation and to look for immediate complications. All procedures were performed by two of four interventional radiologists, all with more than 5 years of experience in thermal ablation procedures.

All procedures were performed in the Interventional Radiology department under general anaesthesia (performed following usual protocols); in all cases, it was possible to perform ablation as preoperatively planned. No minor or major complications were detected. After laser ablation, all patients were observed overnight and discharged the following day. All three nodules were completely ablated with no evidence of residual enhancement in contrast-enhanced CT examinations at the 6 weeks (100% technical success). Contrast-enhanced CT performed at 12 months showed complete ablation with no local tumour progression. All contrast-enhanced CT scans were acquired using triple-phase protocols, according to usual practice.

**Fig. 1:**
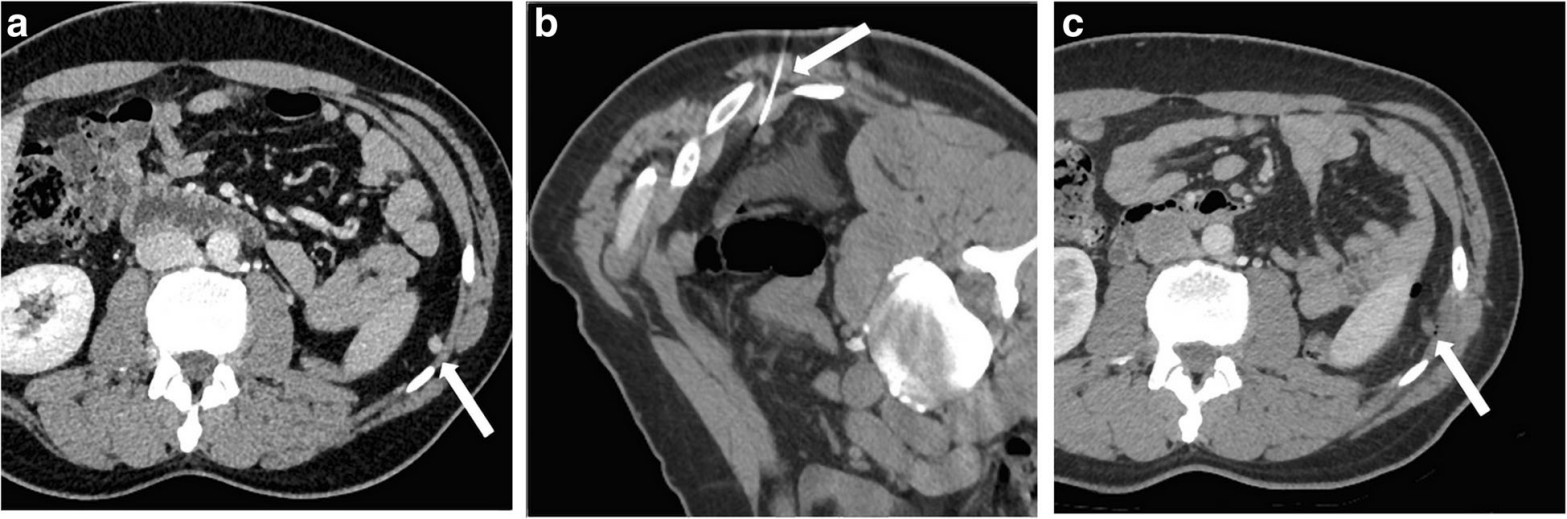
Forty-two-year-old male patient with small left recurrence of renal cell carcinoma. **a** Contrast-enhanced computed tomography (CT) scan before treatment. A pathological nodule of 8 mm is clearly visualised in the left kidney fossa (white arrow). **b** Unenhanced CT scan with the patient placed in a prone position. Laser ablation procedure: the needle of the laser device is clearly seen placed insight the pathological nodule during the treatment (white arrow). **c** Contrast-enhanced CT scan after treatment. The small pathological nodule does not show any contrast uptake demonstrating the effectiveness of the treatment (white arrow)

A case of a patient with an 8-mm recurrence located in renal fossa is presented in Fig. 1. Table 2 reports characteristics of the recurrent lesions (number, size, location, timing), details of the procedure and of the immediate postoperative period (time, energy used, need of hydrodissection, hospital days, complication), and results of follow-ups.

**Table 2:** Details of recurrent tumour, procedure, and follow-up

	Patients		
	1	2	3
Time to recurrence (months)	49	72	30
Lesion number	1	1	1
Tumour recurrence size (mm)	8	12	6
Tumour recurrence location	Renal fossa	Perisplenic	Adrenal loggia
Procedure time (min)	70	65	46
Energy used (J)	1,800	1,800	1,200
Hydrodissection	Yes	No	No
Hospital stay (days)	2	2	2
Complications	None	None	None
Follow-up (CT at < 2 days)	Negative	Negative	Negative
Follow-up (CT at 6 weeks)	Negative	Negative	Negative
Follow-up (CT at 6 months)	Negative	Negative	Negative

*CT* Computed tomography

## Discussion

We retrospectively reviewed the cases of three male patients affected with abdominal recurrent RCC treated with laser ablation. We included all patients who, after undergoing previous treatments for RCC (surgery or image-guided thermal ablation), had a local recurrence of disease with lesions smaller than 2 cm and no other metastatic site elsewhere after staging with a contrast-enhanced CT scan.

Recurrences of RCC after surgical treatment are rare but, when they occur, are associated with a negative prognosis; although surgery remains the gold standard, reintervention not only increases morbidity for the patient but is also not always feasible. Some patients may not undergo surgery because of advanced age, end-stage renal disease, and other comorbidities. Furthermore, postoperative fibrosis, altered anatomy from prior surgery, and recurrent disease adjacent to retroperitoneal structures can cause anatomical limitations that make complex a complete resection of the recurrence lesion [16, 17].

Percutaneous ablative therapies, thanks to the use of an image-guided approach and their minimally invasive nature, could bypass these limits and could represent an effective and less invasive therapeutic strategy in patients with recurrent RCC. However, experiences reporting image-guided thermal ablation of recurrent RCCs are still limited [9–11] and deal mainly with the use of RFA.

The first case was reported in 2002 by McLaughlin et al. [10]. They described the use of percutaneous RFA after radical nephrectomy for a patient with recurrent disease considered non-resectable due to the close proximity to the abdominal aorta. The intervention was successful and without any complications; the patient remained disease-free for the next 16 months of follow-up. The safety and oncological efficacy of this procedure were further proved by Monfardini et al. [9], with their eight patients who were treated with percutaneous RFA for local recurrence after surgery (radical or partial nephrectomy). No complication was reported, and all nodules (mean size = 1.6 cm) were completely ablated. After the procedure, for a mean follow-up of 12 months, no residual disease has been observed.

Recently, Zhou et al. [11] have studied a cohort of 11 patients with local RCC relapses with mean size of 2.8 cm treated using RFA, cryoablation, or, in one case, microwaves. Technical success was achieved in 100% of the cases without major complications. The results have also been promising from an oncological perspective. In all cases, except one, a complete response has been obtained according to RECIST criteria [18], with a local progression-free during the mean follow-up time of 2.5 years. Thus, so far, no established technique has been fully validated for the treatment of recurrent RCC.

At our department, we decided to treat small recurrent RCC (size 6, 8, and 12 mm) with a laser ablation in order to minimise the treatment invasiveness. Laser technology is based on the precise focal application of a laser light through a small optic fibre to achieve a local temperature increase and cell coagulative necrosis [19]. The application through such small applicators is possible thanks to two distinctive features of the laser light beam: collimation and monocromaticity. Accurate image guidance is crucial for a precise and effective thermal ablation, and particularly, the availability of both ultrasound and CT guidance methods, with also the possibility of performing fusion imaging and virtual navigation, might increase the result of ablation of recurrences of RCC, which are often of small dimension and located in difficult anatomical positions [20, 21].

In our small series, laser ablation was proven to be feasible and safe in the treatment of patients with recurrent RCCs, without complications, and with excellent recovery times (see Table 2). To the best of our knowledge, this paper represents the first description of the application of laser ablation to recurrent RCC.

In conclusion, this study, despite the limitations, underlines the feasibility and the potential curative role of laser ablation for small isolated RCC local recurrence after surgery. Such treatment option holds the potential to offer a minimally invasive effective treatment to patients with recurrence after RCC surgical resection. Larger studies with longer follow-up are needed to confirm these preliminary results, possibly comparing laser ablation to surgical re-interventions or other thermal ablation modes.

## Data Availability

The relevant data have been included in the manuscript. The datasets used and/or analysed during the current study are available from the corresponding author on reasonable request.

## Abbreviations

CT: Computed tomography
RCC: Renal cell carcinoma
RFA: Radiofrequency ablation
